# Reproductive barriers in *Serapias x kelleri*

**DOI:** 10.1186/s40529-025-00477-9

**Published:** 2025-09-30

**Authors:** Francesco Carlomagno, Giuseppe Pellegrino

**Affiliations:** Department of Biology, Ecology and Earth Sciences, University of Calabria, 87036 Rende, CS Italy

**Keywords:** Hybrid sterility, Mediterranean orchids, Postzygotic isolation, Reproductive barriers, *Serapias x kelleri*

## Abstract

**Background:**

Understanding the reproductive barriers in natural plant hybrids is fundamental for comprehending mechanisms of speciation and evolutionary divergence. Most studies focus on reproductive barriers and gene exchange material between parental species, whereas fewer emphasize the biological significance of hybrid viability and their ability to interbreed with each other or with the parental species. To address this gap, the present study focuses on the hybrid *Serapias x kelleri* and its parental species, *Serapias vomeracea* and *Serapias cordigera* to evaluate reproductive success and the role of prezygotic and postzygotic barriers. Specifically, the purpose of this study was to investigate and evaluate the reproductive success, the biological and evolutionary implications of *Serapias x kelleri*, in terms of fruit production and seeds produced through hand pollination of any possible bidirectional cross combinations between hybrid plants and both parental species.

**Results:**

Controlled manual crosses between hybrids and parental species to assess fruit and viable seed production were conducted. Results revealed no prezygotic barriers, as all pollinated flowers formed fruit. However, postzygotic barriers were evident, with significant variability in viable seed production. Hybrid self-crosses showed the lowest seed viability (0.6%), indicating strong postzygotic isolation and possible inbreeding depression. Crosses between hybrids and parental species exhibited a variable embryo production, with average values ​​ranging from 5,6% to 29,1%. Remarkable is the fact that when the hybrid acts as a pollen donor, the percentage of viable seeds with embryos is higher, with average values ​​ranging from 18.9% to 29.1%. Opposite, when the hybrid receives pollen from one of the parental species, the values ​​of viable seeds decrease dramatically, with averages that do not exceed 10%. These data clearly show an asymmetric reproductive capacity of the hybrid, with significantly higher success when acting as the pollen parent compared to the maternal parent.

**Conclusions:**

This study emphasizes the critical role of reproductive barriers in maintaining species integrity within the *Serapias* genus. Natural hybrids such as *S. x kelleri* can facilitate gene flow between parental species. However, strong postzygotic barriers significantly limit their fertility, thereby driving evolutionary divergence and preserving the genetic identity of each species. These barriers consequently reduce the likelihood of speciation.

**Supplementary Information:**

The online version contains supplementary material available at 10.1186/s40529-025-00477-9.

## Background

Biologists and evolutionists are fascinated by speciation: understand the mechanisms which governing that and comprehend the reproductive barriers that prevent hybridisation between sympatric species are the focus of numerous studies in last years (Coyne and Orr 2004).

A plant species is a group of interbreeding natural populations that are reproductively isolated from other such groups (Dobzhansky 1937; Mayr 1942; Coyne and Orr 2004) preserving their genetic identity by means of pre-pollination and/or post-pollination mechanisms (Rieseberg et al. 2007; Widmer et al. 2009; Wang and Filatov 2023). The first one act on pollen-stigma, pollen-ovule and male–female gametophyte interactions; instead, the post-pollination mechanisms are represented by seed and embryo mortality, hybrid unviability or sterility and ecological inferiority of hybrids (Ramsey et al. 2003; Moccia et al. 2007; Pellegrino et al. 2010).

In several cases, species which live in the same areas evolve species-specific close relationships with pollinators over the time; this is a pre-zygotic reproductive barrier to avoid pollination between sympatric species and in which phenological flowering rhythms overlap (Van der Pijl & Dodson 1966; Dressler 1993). However, deceptive orchids generally offer no reward to pollinators, and they have a low specificity of pollinators (Neiland and Wilcock 1999; Cozzolino et al. 2005). In these orchids the frequency of hybridization events is moderate to high (Cozzolino and Widmer 2005) and have weak pre-mating isolation but strong post-mating isolation (Scopece et al. 2007, 2008).

Hybridization among deceptive orchids, particularly those from the Mediterranean region, has been thoroughly documented (Cozzolino et al. 2006) due to sympatric occurrence, overlapping flowering periods and pollinator sharing (Cozzolino et al. 2005).

Among the Mediterranean orchids, members of the genus *Serapias* are characterized by an unrewarding pollination strategy, with the typical tubular corolla being visited by pollinators as a resting or sleeping place (Dafni et al. 1981; Pellegrino et al. 2005a; Lanzino et al. 2023), and grow in mixed populations, bloom in the same period and share the same pollinator assemblages, favouring the hybrid formation (Baumann and Künkele 1989; Delforge 2005). Hybrids between the two species have been described as *S.* x *kelleri* Camus (Camus 1936; Pellegrino et al. 2010).

Many studies have focused attention on reproductive barriers and gene exchange between parental species (Schumer et al. 2015) but few papers discuss the hypothesis on the biological significance and/or evolutionary potential trajectories of plant hybrid zones (Campbell et al. 2003; Martin et al. 2005; Luca et al. 2012), especially in terms of the viability of the hybrids and their ability to interbreed with each other and/or with the parental species.

The aim of the present study was to understand and evaluate the reproductive successful and the biological and evolutionary significance of the *Serapias x kelleri*, in terms of fruit production and seeds produced by hand pollination of any possible bidirectional cross combinations between hybrid plants and both parental species.

## Materials and methods

### Studied species and site

The genus *Serapias* L. is essentially widespread in the Mediterranean area and its species are characterized by a common floral morphology, three sepals, two lateral petals and hypochile (the proximal part of the labellum) form a structure like a tunnel (tubular corolla). The epichile (the distal part of the labellum) slopes downward and is often pubescent (Barone Lumaga et al. 2012). It has been assumed that floral tube size and shape limit access to the flowers and thus may act as a barrier to gene flow between different *Serapias* species (Pellegrino et al. 2005b). The flowers do not show spur or nectar, have a pollinarium with pollen packed into two pollen masses, and produce olfactory signals (Pellegrino et al. 2012). Almost all species show a unique pollination strategy called shelter imitation: the insects using the floral tube of orchids as a shelter during night, in windy and rainy weather (Gumprecht 1977) or for thermoregulation (Dafni et al. 1981; Felicioli et al. 1998; Lanzino et al. 2023). Only *Serapias lingua*, seems to have evolved a different pollination strategy like sexual deception typical of the genus *Ophrys* (Vereecken et al. 2010; Pellegrino et al. 2017).

In the present study, *Serapias x kelleri* Camus and the parental species, *Serapias vomeracea* (Burm. f.) Briq. and *Serapias cordigera* L., were used. The two parental species differ in floral size (Baumann and Künkele 1989), in terms of width of the epichile (8–13 × 18–30 mm in *S. vomeracea* and 10–14 × 17–24 mm in *S. cordigera*) (Delforge 2005). The two species may be easily discriminated by the epichile, which is typically heart-shaped and dark-brown color in *S. cordigera*. The chromosome number of both species is 2*n* = 36 (D’Emerico et al. 2000).

*S. cordigera* and *S. vomeracea* are widespread species, growing in arid meadows, abandoned agricultural lands, garigues and bushy environments up to 1000 m a.s.l., overlap their phenological flowering periods and the hybridisation between them is possible and reported (Guenther Blaich. http://www.guenther-blaich.de. Accessed 15 October 2024) (Sardaro et al. 2012; Bellusci et al. 2010).

The main pollinators of both species are hymenopteran insects of four genera (*Ceratina*, *Eucera*, *Osmia* and *Tetralonia*) of the superfamily Apoidae (Dafni et al. 1981; Felicioli et al. 1998; Pellegrino et al. 2005a).

Two study areas have been identified in southern Italy (Calabria region), the first in the municipality of Mangone on abandoned agricultural calcareous soil, the second in the municipality of Cassano allo Ionio in an area called Parco del Monte, specifically in a relatively neglected portion of the park.

### Plant material collection

To carry out this study plants belonging to *Serapias vomeracea*, *Serapias cordigera* and its natural hybrid *Serapias* x *kelleri* were used. Collection of individuals was done in compliance with institutional, national, and international guidelines for biodiversity conservation. The studied species do not require special permits for the collection of their vegetative parts, as they are not included in lists of protected species at the national or international level and are not subject to specific regulations at the collection sites.

Moreover, all activities were conducted in accordance with the IUCN Policy Statement on Research Involving Species at Risk of Extinction and the Convention on International Trade in Endangered Species of Wild Fauna and Flora (CITES), where applicable.

### Hand pollination and reproductive success

To carry out the experimental crosses, 5 plants at bud stage for each site for each taxon (*S. vomeracea*, *S. cordigera* and their hybrid, *S. x kelleri*.) have been transplanted into the field and subsequently transferred to a controlled environment in the Plant Biosystematics Laboratory of the University of Calabria.

The crosses were performed manually, using a small tweezer, by removing the pollinarium and placing it on the stigma of a flower from another plant. The positional choice of the flowers to be pollinated on the plant was random and the crossing scheme is shown in Table 1.

**Table 1:** Crossing scheme of the experiments

Artificial crosses			NF	FP	% F	NS	SE	%E	%G	RIpre	RIpost
										HSf	HSs
*S. x kelleri*	X	*S. vomeracea*	16	16	100	11,002	2079	18.89	95.10	0	0.81
*S. x kelleri*	X	*S. cordigera*	16	16	100	11,353	3301	29.08	94.25	0	0.71
*S. vomeracea*	X	*S. x kelleri*	6	6	100	3266	179	5.48	96.50	0	0.94
*S. cordigera*	X	*S. x kelleri*	6	6	100	5337	534	10.00	94.35	0	0.90
*S. x kelleri*	X	*S. x kelleri*	10	10	100	4299	25	0.58	94.10	0	0.99

Fruit set, seeds with embryo, reproductive isolation (i.e. hybrid sterility) in artificial backcrosses between *Serapias x kelleri* and their parental species (*S. vomeracea* and *S. cordigera*), and between hybrid generation. [In each cross, the first species listed is the pollen parent and the second species listed is the pod (seed) parent; *NF* number of flowers observed, *FP* number of fruits produced, *%F* percentage of fruits produced, *NS* number of seeds observed, *SE* number of seeds with embryo, *%E* percentage of seeds with embryo, *%G* percentage of seed germination, *RIpre* Reproductive Isolation at pre-zygotic level (i.e. Hybrid Sterility at fruit production stage), *RIpost* Reproductive Isolation at post-zygotic (i.e. Hybrid Sterility at seed production stage)]

The development of the fruits, following the cross-pollination, was monitored until their collection, before the dehiscence and each mature capsule was stored in an Eppendorf tube and catalogued, in accord to the type of cross and the plant from which it was collected.

The ratio, given by the number of pollinated flowers to the number of fruits formed from them, was used to assess the existence and intensity of prezygotic reproductive barriers.

Subsequently, to evaluate the intensity and existence of postzygotic reproductive barriers, at least 1000 seeds were taken from each capsule, and were observed under an optical microscope with 100 × enlargement to assess the ratio between seeds with an embryo and those without.

Successively, seeds with an embryo were kept at 4 °C until the sowing procedure, and tested for their asymbiotic germination capacity (Pellegrino 2016), to determine the strength of late postzygotic barriers. Seeds were sterilized in a 1:10 Clorox solution (0.5% available chlorine) for 7 min, washed with sterile distilled water, then spread over BM1 medium (Van Waes and Derbergh 1986) in Petri dishes and kept in the dark in a growth chamber at 25 °C. Approximately 200 seeds were sown in triplicate for each cross treatment. Germination was considered to have occurred when the testa of the enlarging embryo split out of the seed. The total proportion of germinating seeds was calculated when no further germination occurred for up to 2 months after sowing at the latest.

We quantified the pre-zygotic reproductive isolation (RIpre) and hybrid sterility at fruit production stage (HSf) of *S. x kelleri* by performing manual crossing experiments between hybrids and their parental species following the form:

RIpre-zygotic (= HSf) = 1 − [number of fruits produced/number of pollinated flowers].

Post-zygotic reproductive isolation (RIpost) and hybrid sterility at viable seed production stage (HSs) were defined as the number of viable embryos (i.e., viable seeds) produced. That is:

RIpost-zygotic (= HSs) = 1 – [number of viable seeds/total number of counted seeds].

Values of these indices vary from zero (no isolation, no hybrid sterility) to one (total isolation, hybrid sterility) (Moyle et al. 2004; Pellegrino et al. 2010).

### Statistical analysis

To evaluate differences in viable seed production among cross types, we used Fisher’s exact test to compare the proportions of embryo-containing seeds for each pairwise combination of crosses. Additionally, Chi-squared tests were conducted to confirm significant differences across all crosses.

All statistical analyses were performed using PAST software package (version 4.03; Hammer et al. 2001), and results were considered statistically significant when p < 0.05.

## Results

In all the crosses performed, the pollinated flowers formed capsules (100%), indicating no prezygotic barriers. This is particularly relevant for the hybrid *Serapias x kelleri* which produced capsules in all crosses.

The mean percentage of seeds with embryos significantly differs across cross combinations.

The summary statistics (Table 1) highlights significant variability in post-zygotic reproductive success across different manual cross combinations. In *S. x kelleri* X *S. vomeracea* the percentage of seeds with embryos shows considerable fluctuations (from 0.3% to 38.4%), with an average value of 18.89% that suggesting a degree of reproductive compatibility but with limitations in hybrid viability. Similarly, crosses of *S. x kelleri* with *S. cordigera* reveal higher embryo production in certain capsules (1.9% to 66.4%) with an average value of 29.08%.

Crosses between *S. vomeracea* X *S. x kelleri* show consistently low embryo rates across observations (0% to 11.8%), with an average value of 5.48%. This suggests stronger postzygotic barriers when *S. vomeraceae* contributes as the pollen donor and probably limiting hybrid viability.

The cross-type *S. cordigera* X *S. x kelleri* produces slightly higher embryo rates than *S. vomeracea* X *S. x kelleri*, though still low (3.2–19.1%), with an average value of 10.00%, indicating high level of reproductive isolation.

The embryo rate of self-crosses of *S. x kelleri* remains under 1% consistently across observations (0% to 1.0%) with an average value of 0.58%, and highest value of hybrid sterility (0.99%). This remarkably low viability suggests strong postzygotic barriers and likely inbreeding depression limiting embryo viability.

Pairwise statistical comparisons definitely confirmed the high significance of these differences in viable seed production between cross types. For instance, the hybrid self-cross (*S. x kelleri* X *S. x kelleri*) showed a significantly lower proportion of viable seeds when compared to crosses involving parental specie.

These significant results confirm that hybrid fertility is asymmetrically reduced, and that the reproductive success of the hybrid significantly depending on the cross direction, underscoring the complex nature of reproductive isolation in *Serapias*. A full list of all pairwise comparisons and their respective statistical values, including Chi-squared values, Fisher's exact p-values and significance levels (*** for p < 0.001), is reported in Table 2. Seed germinability of all hand pollination experiments ranged from 94 to 96.5%, indicating that, although only a small proportion of seeds contained embryos, those embryos had a high probability of germination.

**Table 2:** Statistical significance of embryo-containing seed production across different cross combinations, as determined by Fisher's exact test and Chi-squared test (df indicates degrees of freedom)

Comparison (Type of Cross 1 vs Type of Cross 2)	χ^2^ (df = 1)	Fisher’s Exact p-value	Significance
*S. x kelleri* X *S. x kelleri ****vs**** S. x kelleri* X *S. cordigera*	1512	< 0.0001 (0)	***
*S. x kelleri* X *S. kelleri ****vs**** S. x kelleri* X *S. vomeracea*	871.72	< 0.0001 (3.22*10^–278^)	***
*S. x kelleri* X *S. kelleri ****vs**** S. cordigera* X *S. kelleri*	384.39	< 0.0001 (7.59*10^–107^)	***
*S. x kelleri* X *S. vomeracea ****vs**** S. x kelleri* X* S. cordigera*	318.35	< 0.0001 (1.13*^10–71^)	***
*S. vomeracea* X *S. kelleri ****vs**** S. cordigera* X *S. kelleri*	50.80	< 0.0001 (3,10*10^–13^)	***

Asterisks indicate statistical significance levels: *p < 0.05, **p < 0.01, ***p < 0.001. All comparisons show a significance of p < 0.0001

**Fig. 1:**
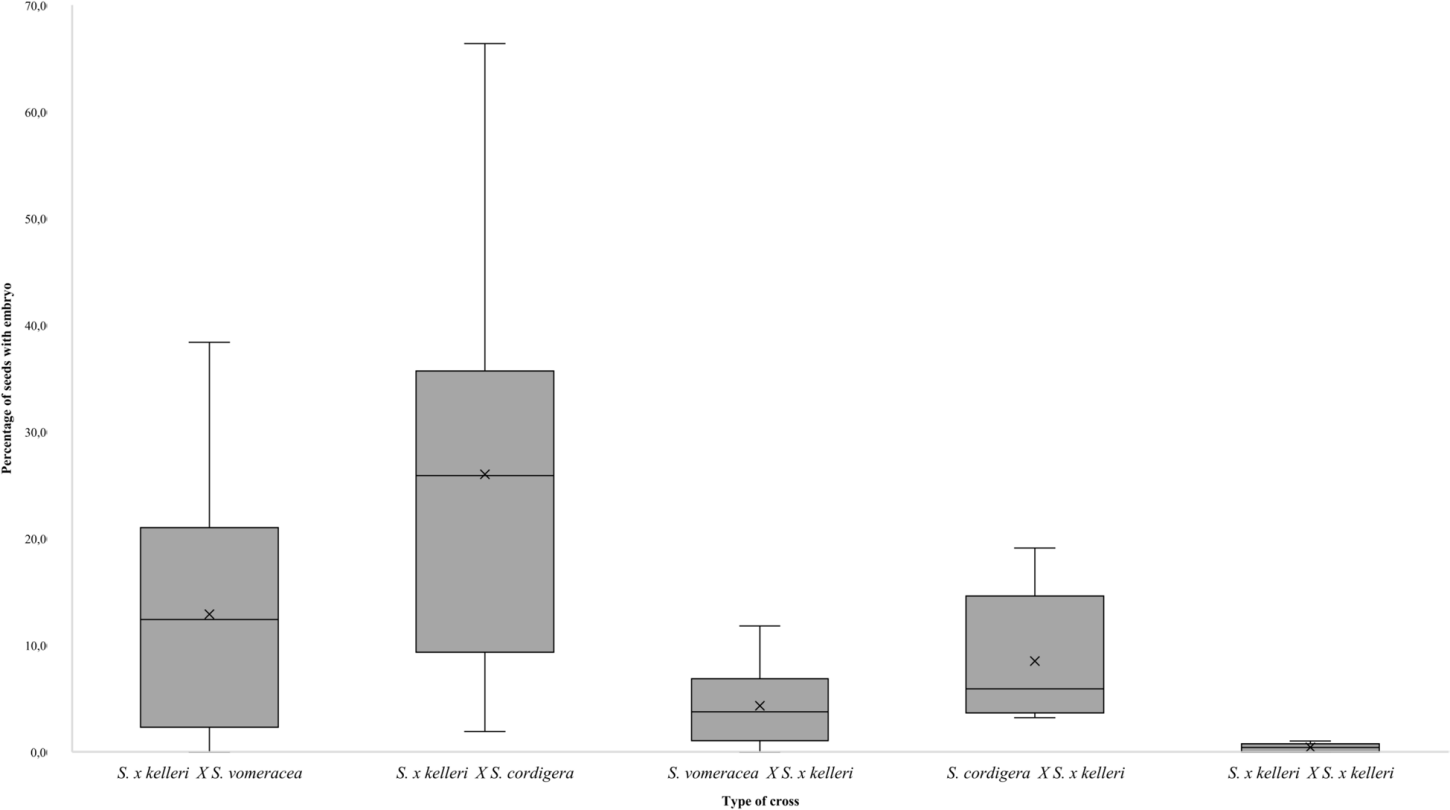
Percentage of embryo containing seeds per capsule. Differences in reproductive success per capsule among the following crosses: *S. x kelleri* X *S. vomeracea*, *S. x kelleri* X *S. cordigera*, *S. vomeracea* X *S. x kelleri*, *S. cordigera* X *S. x kelleri* and *S. x kelleri* X *S. x kelleri*

The data show the substantial differences in reproductive success among the different crosses (Fig. 1), some crosses produce a very low percentage of seeds with embryos, suggesting the presence of postzygotic reproductive barriers. The two crosses *S. x kelleri* X *S. vomeracea* and *S. x kelleri* X *S. cordigera* demonstrated a reasonable postzygotic reproductive success with the production of seeds containing embryos; however, the presence of seeds without embryos suggests the existence of postzygotic reproductive barriers that impede or limit the development of hybrids. In the intraspecific crosses (*S. x kelleri* X *S. x kelleri*), the percentage of seeds with embryos is very low. This may indicate a form of inbreeding depression among hybrids, leading to greater difficulty in obtaining viable seeds when there is a cross of hybrid individuals.

The results suggest that significant postzygotic barriers exist that influence reproductive success in terms of producing seeds with embryos. The presence of strong postzygotic barriers is a key indicator of reproductive isolation between these species and may help explain the low rate of natural hybridization, despite the sympatry and overlap in flowering periods, thus limiting the possibility of forming fertile hybrids.

**Fig. 2:**
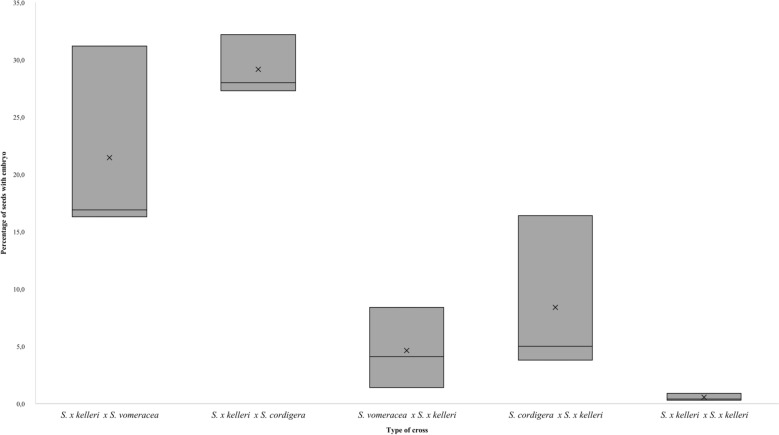
Percentage of embryo containing seeds per plant. Differences in reproductive success per plant among the following crosses: *S. x kelleri* X *S. vomeracea*, *S. x kelleri* X *S. cordigera*, *S. vomeracea* X *S. x kelleri*, *S. cordigera* X *S. x kelleri* and *S. x kelleri* X *S. x kelleri*

The percentage of seeds containing embryos per individual plant, a marked variability in post-zygotic reproductive success, is observed among the examined *Serapias* species (Fig. 2). Notably, the hybrid *S. x kelleri* shows lower rates of embryo-containing seed production compared to the parental species, indicating reduced reproductive efficacy. The observed differences in reproductive success rates per plant suggest that, while crosses between *S. x kelleri* and the parental species are possible, the hybrid still faces limitations. This result also supports the hypothesis that postzygotic barriers exist at an individual level, which restrict gene flow and help maintain reproductive isolation among the species.

**Fig. 3:**
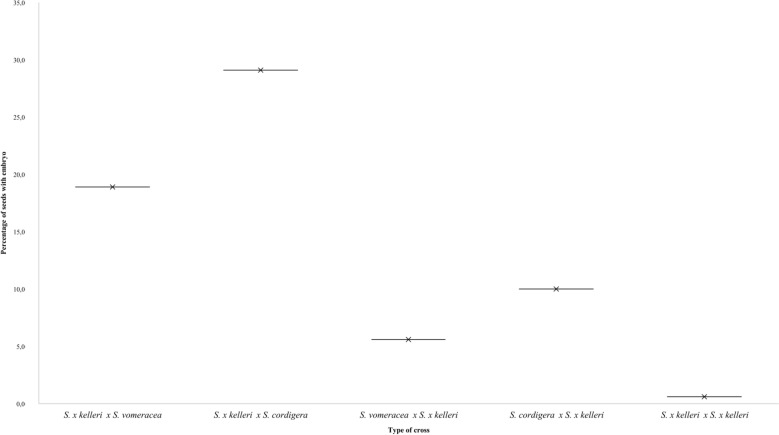
Percentage of embryo containing seeds per species. Differences in reproductive success per species among the following crosses: *S. x kelleri* X *S. vomeracea*, *S. x kelleri* X *S. cordigera*, *S. vomeracea* X *S. x kelleri*, *S. cordigera* X *S. x kelleri* and *S. x kelleri* X *S. x kelleri*

An overall comparison among the studied *Serapias* species (Fig. 3) highlights that *S. cordigera* and *S. vomeracea* have a significantly higher capacity to produce viable seeds compared to the hybrid *S. x kelleri*. The low percentages of embryo-containing seeds observed in the hybrid further indicate the presence of strong postzygotic reproductive barriers, suggesting reduced fertility that makes the formation of persistent, fertile hybrid populations unlikely.

Our data shows a complete absence of isolation at fruit production stage, while high levels of isolation, and so hybrid sterility were observed at viable seed production stage (Table 1).

## Discussion

In this study, manual crossbreeding experiments enabled us to evaluate reproductive success in terms of fruit and viable seed production with embryos in the natural hybrids of *Serapias x kelleri*, as compared to its parental species. The research highlights the various roles natural orchid hybrids can play and the function of postzygotic reproductive barriers as natural mechanisms that restrict hybridization among sympatric *Serapias* species. This promotes evolutionary divergence within the genus and preserves the genetic integrity of the species.

The low percentage of viable seeds with embryos observed in hybrid crosses, compared to the fertility rates of the parental species, suggests that natural hybridization minimally contributes to gene flow, while reinforcing reproductive isolation. Although natural hybridization can be an important evolutionary mechanism in plants (Rieseberg 1997), it often serves as an effective postzygotic barrier (Campbell et al. 2003; Martin et al. 2005). The hypothesis that the high frequency of natural hybrids and hybrid zones in Mediterranean orchids may play a significant role in speciation and evolution within this group (Ehrendorfer 1980; Van der Pijl & Dodson 1966) remains inconclusive.

Research into the genus *Serapias* remains complex. In bidirectional crosses, where *S. x kelleri* acted as both pollen donor and recipient with its parental species (*S. cordigera* and *S. vomeracea*), consistent fruit production was observed (see Supplementary Material). This suggests that the hybrid could serve as a genetic bridge between the two species, leading to frequent and numerous exchanges of genetic material. Our findings are well-supported by previous molecular studies, which revealed a high presence of hybrids resulting from backcrossing (introgression) or second-generation hybrids in a contact zone in northern Calabria between *S. cordigera* and *S. vomeracea* (Pellegrino et al. 2010).

However, the reduced percentage of seeds with embryos in certain crosses could represent a natural barrier limiting hybridization among sympatric species, thus fostering evolutionary divergence. The data obtained support the hypothesis that, although hybridization may occur in nature, selection against less viable hybrids, particularly those resulting from crosses within the same hybrid population, could represent a significant postzygotic barrier (Moccia et al. 2007; Pellegrino et al. 2010).

Indeed, the average percentage of viable seeds with embryos in hybrids crossed with parental species never exceeds 30%, fluctuating between a minimum of 5.6% and a maximum of 29.1% (Fig. 3). When compared to intraspecific crosses, it is evident that the percentage of viable seeds is drastically lower. Previous studies have shown embryo-bearing seed rates of 87.5% for *S. cordigera* and 92.0% for *S. vomeracea* (Bellusci et al. 2009).

Despite high fruit production rates in these crosses, the low presence of viable seeds suggests that the hybrid indeed acts as a partial barrier to gene flow between the two species. This implies the presence of strong postzygotic barriers and potential inbreeding depression, contributing to the low viability of seeds produced by individuals of the same hybrid population. Selective postzygotic barriers, in effect, influence hybrid fertility and act as reproductive isolation mechanisms, limiting gene flow and favouring speciation within the *Serapias* genus (Christie et al. 2022).

Interestingly, the bidirectional crosses revealed differences: when the hybrid acts as a pollen donor, the percentage of viable seeds with embryos is higher, with mean values between 18.9% and 29.1% and individual observations exceeding 50% (see Supplementary Material). In contrast, when the hybrid receives pollen from one of the parental species, viable seed values decrease sharply, with averages not exceeding 10% and individual observations never exceeding 20% (see Supplementary Material). These data clearly show a greater reproductive capacity of the hybrid depending on whether it contributes to the maternal or paternal lineage.

It remains to be determined whether *S. x kelleri* could represent an actual case of speciation. Although crosses between hybrid individuals result in 100% fruit formation, the percentage of seeds with embryos is nearly zero (0.6%), leading to the reasonable conclusion that reproductive success in crosses between hybrids is practically null. This excludes the likelihood that *S. x kelleri* is progressing toward speciation. However, considering the hybrid's limited fertility, one could hypothesize that if favourable environmental conditions arise, the hybrid might adapt to new conditions, increase its reproductive success, and potentially become a new species. The hybrid between *S. vomeracea* and *S. cordigera* indeed shows the potential to initiate a speciation event through hybridization.

## Supplementary Information

Additional file 1.

## Data Availability

All data generated or analysed during this study are included in this published article [and its supplementary information files].
