# Supplementary material for: Reproductive barriers in Serapias x kelleri
Source: Bot Stud. 2025 Sep 30;66:32. doi: 10.1186/s40529-025-00477-9 (PMC12484518; doi:10.1186/s40529-025-00477-9)
Supplement: Supplementary file 1 — Additional file 1. [file 40529_2025_477_MOESM1_ESM.docx]

**Supplementary material**

Table 3. The ratio of seeds with embryos to seeds without embryos analyzed in each capsule

| **Type of cross** | **Total seeds analyzed per capsule** | **Seeds with embryo** | **Seeds without embryo** | |
| --- | --- | --- | --- | --- |
| *S. x kelleri*  X *S. vomeracea* | 738 | 2 | 736 |  |
| *S. x kelleri*  X *S. vomeracea* | 882 | 646 | 236 |  |
| *S. x kelleri* X *S. vomeracea* | 732 | 106 | 626 |  |
| *S. x kelleri* X *S. vomeracea* | 870 | 53 | 817 |  |
| *S. x kelleri* X *S. vomeracea* | 1.068 | 171 | 897 |  |
| *S. x kelleri* X *S. vomeracea* | 772 | 60 | 712 |  |
| *S. x kelleri* X *S. vomeracea* | 622 | 14 | 608 |  |
| *S. x kelleri* X *S. vomeracea* | 609 | 14 | 595 |  |
| *S. x kelleri* X *S. vomeracea* | 0 | 0 | 0 |  |
| *S. x kelleri* X *S. vomeracea* | 183 | 0 | 183 |  |
| *S. x kelleri* X *S. vomeracea* | 714 | 87 | 627 |  |
| *S. x kelleri* X *S. vomeracea* | 754 | 143 | 611 |  |
| *S. x kelleri* X *S. vomeracea* | 748 | 94 | 654 |  |
| *S. x kelleri* X *S. vomeracea* | 674 | 178 | 496 |  |
| *S. x kelleri* X *S. vomeracea* | 935 | 359 | 576 |  |
| *S. x kelleri* X *S. vomeracea* | 701 | 152 | 549 |  |
| *S. x kelleri* X *S. cordigera* | 1.135 | 345 | 790 |  |
| *S. x kelleri* X *S. cordigera* | 593 | 132 | 461 |  |
| *S. x kelleri* X *S. cordigera* | 108 | 2 | 106 |  |
| *S. x kelleri* X *S. cordigera* | 208 | 40 | 168 |  |
| *S. x kelleri* X *S. cordigera* | 814 | 282 | 532 |  |
| *S. x kelleri* X *S. cordigera* | 948 | 629 | 319 |  |
| *S. x kelleri* X *S. cordigera* | 899 | 369 | 530 |  |
| *S. x kelleri* X *S. cordigera* | 852 | 74 | 778 |  |
| *S. x kelleri* X *S. cordigera* | 713 | 61 | 652 |  |
| *S. x kelleri* X *S. cordigera* | 688 | 77 | 611 |  |
| *S. x kelleri* X *S. cordigera* | 704 | 101 | 603 |  |
| *S. x kelleri* X *S. cordigera* | 971 | 341 | 630 |  |
| *S. x kelleri* X *S. cordigera* | 672 | 26 | 646 |  |
| *S. x kelleri* X *S. cordigera* | 754 | 401 | 353 |  |
| *S. x kelleri* X *S. cordigera* | 622 | 223 | 399 |  |
| *S. x kelleri* X *S. cordigera* | 672 | 198 | 474 |  |
| *S. vomeracea* X *S. x kelleri* | 771 | 91 | 680 |  |
| *S. vomeracea* X *S. x kelleri* | 664 | 29 | 635 |  |
| *S. vomeracea* X *S. x kelleri* | 614 | 32 | 582 |  |
| *S. vomeracea* X *S. x kelleri* | 617 | 19 | 598 |  |
| *S. vomeracea* X *S. x kelleri* | 0 | 0 | 0 |  |
| *S. vomeracea*  X *S. x kelleri* | 561 | 8 | 553 |  |
| *S. cordigera* X *S. x kelleri* | 1.102 | 144 | 958 |  |
| *S. cordigera* X *S. x kelleri* | 1.332 | 254 | 1067 |  |
| *S. cordigera* X *S. x kelleri* | 782 | 30 | 752 |  |
| *S. cordigera* X *S. x kelleri* | 743 | 24 | 719 |  |
| *S. cordigera* X *S. x kelleri* | 704 | 34 | 670 |  |
| *S. cordigera* X *S. x kelleri* | 685 | 48 | 637 |  |
| *S. x kelleri* X *S. x kelleri* | 636 | 6 | 630 |  |
| *S. x kelleri* X *S. x kelleri* | 586 | 6 | 580 |  |
| *S. x kelleri* X *S. x kelleri* | 534 | 4 | 530 |  |
| *S. x kelleri* X *S. x kelleri* | 587 | 2 | 585 |  |
| *S. x kelleri* X *S. x kelleri* | 230 | 0 | 230 |  |
| *S. x kelleri* X *S. x kelleri* | 628 | 3 | 625 |  |
| *S. x kelleri* X *S. x kelleri* | 103 | 0 | 103 |  |
| *S. x kelleri* X *S. x kelleri* | 514 | 3 | 511 |  |
| *S. x kelleri* X *S. x kelleri* | 0 | 0 | 0 |  |
| *S. x kelleri* X *S. x kelleri* | 481 | 1 | 480 |  |

Table 4. Ratio of seeds with embryos to seeds without embryos analyzed for each plant

| **Type of cross** | **Total seeds analyzed per capsule** | **Seeds with embryo** | **Seeds without embryo** |
| --- | --- | --- | --- |
| *S. x kelleri* x *S. vomeracea* | 6.293 | 1064 | 5229 |
| *S. x kelleri* x *S. vomeracea* | 3.073 | 501 | 2572 |
| *S. x kelleri* x *S. vomeracea* | 1.636 | 510 | 1126 |
| *S. x kelleri* x *S. cordigera* | 2.858 | 800 | 2058 |
| *S. x kelleri* x *S. cordigera* | 4.804 | 1311 | 3493 |
| *S. x kelleri* x *S. cordigera* | 3.691 | 1189 | 2502 |
| *S. vomeracea* x *S. x kelleri* | 1.435 | 121 | 1314 |
| *S. vomeracea* x *S. x kelleri* | 1.231 | 50 | 1181 |
| *S. vomeracea* x *S. x kelleri* | 561 | 8 | 553 |
| *S. cordigera* x *S. x kelleri* | 2.434 | 399 | 2035 |
| *S. cordigera* x *S. x kelleri* | 782 | 30 | 752 |
| *S. cordigera* x *S. x kelleri* | 2.132 | 107 | 2025 |
| *S. x kelleri* x *S. x kelleri* | 636 | 6 | 630 |
| *S. x kelleri* x *S. x kelleri* | 1.120 | 10 | 1110 |
| *S. x kelleri* x *S. x kelleri* | 587 | 2 | 585 |
| *S. x kelleri* x *S. x kelleri* | 961 | 3 | 958 |
| *S. x kelleri* x *S. x kelleri* | 995 | 4 | 991 |

Table 5. Ratio of seeds with embryos to seeds without embryos analyzed for each species

| **Type of cross** | **Total seeds analyzed per capsule** | **Seeds with embryo** | **Seeds without embryo** |
| --- | --- | --- | --- |
| *S. x kelleri* x *S. vomeracea* | 11.002 | 2079 | 8923 |
| *S. x kelleri* x *S. cordigera* | 11.353 | 3304 | 8049 |
| *S. vomeracea* x *S. x kelleri* | 3.227 | 181 | 3046 |
| *S. cordigera* x *S. x kelleri* | 5.348 | 535 | 4813 |
| *S. x kelleri* x *S. x kelleri* | 4.299 | 26 | 4273 |

|  | **S.xkelleri X**  **S. vomeracea** | | **S.xkelleri X**  **S. vomeracea** | | **S.xkelleri X**  **S. vomeracea** | | **S.xkelleri X**  **S. vomeracea** | | **S.xkelleri X**  **S. vomeracea** | |
| --- | --- | --- | --- | --- | --- | --- | --- | --- | --- | --- |
|  | Seeds with embryo | Seeds without embryo | Seeds with embryo | Seeds without embryo | Seeds with embryo | Seeds without embryo | Seeds with embryo | Seeds without embryo | Seeds with embryo | Seeds without embryo |
| **N** | 16 | 16 | 16 | 16 | 6 | 6 | 6 | 6 | 10 | 10 |
| **Min** | 0 | 0 | 2 | 106 | 0 | 0 | 24 | 637 | 0 | 0 |
| **Max** | 646 | 897 | 629 | 790 | 91 | 680 | 254 | 1067 | 6 | 630 |
| **Sum** | 2079 | 8923 | 3301 | 8052 | 179 | 3048 | 534 | 4803 | 25 | 4274 |
| **Mean** | 129.9375 | 557.6875 | 206.3125 | 503.25 | 29.83333 | 508 | 89 | 800.5 | 2.5 | 427.4 |
| **Std. error** | 41.58229 | 54840.1 | 43.79685 | 49.14337 | 13.20459 | 103.1791 | 37.7103 | 70.37649 | 0.7340905 | 72.67679 |
| **Variance** | 27665.4 | 54840.1 | 30690.63 | 38641.13 | 1046.167 | 63875.6 | 8532.4 | 29717.1 | 5.388889 | 52819.16 |
| **Stand. dev** | 166.3292 | 234.1796 | 175.1874 | 196.5735 | 32.3445 | 252.7362 | 92.37099 | 172.3865 | 2.321398 | 229.8242 |
| **Median** | 90.5 | 609.5 | 165 | 531 | 24 | 590 | 41 | 735.5 | 2.5 | 520.5 |

Table 6. Mean Percentage of Embryo-Containing Seeds Across Different Cross Combinations in *Serapias x kelleri*
